# Flexible Carbon-Use Efficiency across Litter Types and during Decomposition Partly Compensates Nutrient Imbalances—Results from Analytical Stoichiometric Models

**DOI:** 10.3389/fmicb.2017.00661

**Published:** 2017-04-26

**Authors:** Stefano Manzoni

**Affiliations:** ^1^Department of Physical Geography, Stockholm UniversityStockholm, Sweden; ^2^Bolin Centre for Climate Research, Stockholm UniversityStockholm, Sweden

**Keywords:** carbon-use efficiency, C:N ratio, nitrogen mineralization, nitrogen immobilization, stoichiometric model

## Abstract

Mathematical models involving explicit representations of microbial processes have been developed to infer microbial community properties from laboratory and field measurements. While this approach has been used to estimate the kinetic constants related to microbial activity, it has not been fully exploited for inference of stoichiometric traits, such as carbon-use efficiency (CUE). Here, a hierarchy of analytically-solvable mass-balance models of litter carbon (C) and nitrogen (N) dynamics is developed, to infer decomposer CUE from measured C and N contents during litter decomposition. The models are solved in the phase space—expressing litter remaining N as a function of remaining C—rather than in time, thus focusing on the stoichiometric relations during decomposition rather than the kinetics of degradation. This approach leads to explicit formulas that depend on CUE and other microbial properties, which can then be treated as model parameters and retrieved via nonlinear regression. CUE is either assumed time-invariant or as a function of the fraction of remaining litter C as a substitute for time. In all models, CUE tends to increase with increasing litter N availability across a range of litter types. When temporal trends in CUE are considered, CUE increases during decomposition of N-poor litter cohorts, in which decomposers are initially N-limited, but decreases in N-rich litter possibly due to C-limitation. These patterns of flexible CUE that partly compensate stoichiometric imbalances are robust to moderate shifts in decomposer C:N ratio and hold across wide climatic gradients.

## Introduction

Microbial decomposers play a key role in global carbon (C) and nutrient cycles by driving the degradation and mineralization of organic matter. C from decomposing compounds is partly used for growth of new cells and partly respired for energy production. The partitioning of C between these anabolic and catabolic processes has consequences on the rates of C accumulation in soils and sediments, because C allocated to microbial growth may remain in the system, whereas respired C is lost (Six et al., 2006; Sinsabaugh et al., 2013). The decomposer C-use efficiency (CUE), defined as the ratio of decomposer growth rate over the rate of organic matter uptake, integrates these processes into a single parameter (Manzoni et al., 2012b; Geyer et al., 2016). High values of CUE characterize systems where C is effectively retained in new biomass, whereas low values are suggestive of a leaky system. Patterns in CUE thus become useful indicators of trends in potential C storage along environmental and stoichiometric gradients.

Empirically estimating CUE, however, is challenging because this parameter integrates processes occurring at different time scales and carried out by different fractions of the microbial community (Geyer et al., 2016). As a consequence, estimated CUE values are sensitive to the chosen experimental approach (e.g., length of the measurement procedure, addition of labile C), possibly masking its “true” variability caused by ecological and biogeochemical factors (e.g., abiotic factors such as temperature and moisture, community status and composition, nutrient availability). These empirical approaches are mainly based on the definition of CUE as ratio of biomass production over substrate consumption. Rates of microbial production can be measured either directly as change in microbial biomass or indirectly as difference between consumption and respiration. Similarly, consumption rates can be measured directly or indirectly as sum of production and respiration. In any case, the type and amount of C amendment, length of the experiment, and measurement uncertainties affect the estimated CUE values, potentially complicating the comparison and interpretation of results across experiments.

As an alternative and complement to these empirical approaches, CUE could be estimated by fitting analytical models where CUE appears as an explicit parameter to biogeochemical data (pools or fluxes). This approach is conceptually equivalent to inferring microbial traits with kinetic models (Panikov, 1995; Pansu et al., 2004; Manzoni et al., 2012a). Using this rationale, CUE has been estimated along gradients in temperature (Wetterstedt and Ågren, 2011), inorganic nutrient availability (Ågren et al., 2001), and litter elemental composition (Nicolardot et al., 2001; Manzoni et al., 2008a, 2010). Compared to the empirical approaches, these methods exploit coarser data that might be less prone to uncertainties, such as total soil or litter C and nutrient content. The most obvious disadvantage is that they all rely on the simplified mathematical representation of our conceptual understanding of microbial functioning. The main purpose of this contribution is to further develop these analytical methods to reduce their level of simplification and thus offer more realistic estimates of CUE and its trends during decomposition.

These biogeochemical models are based on mass balance equations (as described in Section Theory), which are solved analytically to predict C or nutrient changes through time during decomposition of a cohort of litter. To allow comparison across incubation environmental conditions, the analytical solutions can be expressed in terms of nutrient content as a function of C content instead of time (Ågren and Bosatta, 1987; Manzoni et al., 2008a). This representation removes environmental effects (e.g., temperature and moisture) and compartment size effects (decomposition is faster when there is more C available) that alter the kinetics of decomposition, and focuses on the stoichiometric relations among elements using C content as a proxy of time. Even after removing temporal effects, these models should still account for changes in decomposer community traits as decomposition progresses, but as of now, they all assume time-invariant traits. For example, CUE is expected to vary during decomposition because it is sensitive to changes in nutrient availability and C quality (Cotrufo et al., 2013; Frey et al., 2013; Sinsabaugh et al., 2013). Other traits such as microbial biomass elemental composition might also shift, partly tracking trends in substrate nutrient concentration, thereby reducing stoichiometric imbalances. The microbial P:C ratio is particularly flexible in aquatic bacterial communities grown along wide gradients of P availability (Cotner et al., 2010; Godwin and Cotner, 2015). Also the microbial N:C ratio can increase with widening organic matter N:C in both aquatic and terrestrial systems (Tezuka, 1990; Wagener and Schimel, 1998), but in general decomposer communities can be regarded as nearly homeostatic with respect to N (Cotner et al., 2010; Fanin et al., 2013; Xu et al., 2013).

In this contribution, trends in CUE and other decomposer traits are taken into account in a new set of analytical stoichiometric models. Different from previous approaches that assumed time-invariant CUE (Manzoni et al., 2010; Wetterstedt and Ågren, 2011; Ågren et al., 2013), the aim here is to quantify trends in CUE during decomposition of litter cohorts, and identify the stoichiometric and environmental drivers of such trends. Moreover, results assuming weakly homeostatic decomposer communities are compared to those assuming strict homeostasis, thus providing a complete picture of trait variability effects on decomposition patterns. The analytical models are employed to estimate decomposer traits via nonlinear fitting to litterbag C and N content data. Finally, results are compared to observations and previous CUE estimates using the simpler model with time-invariant traits, and litter quality and climatic conditions are considered as potential drivers of the observed trends.

## Methods

Estimating CUE from decomposition data requires a mathematical model that explicitly includes CUE as a fitting parameter. In Section Theory, a minimal process-model of C and N dynamics during decomposition is presented, and analytical equations suitable for CUE estimation are derived considering: (i) time-invariant decomposer traits (model I, Section Time-Invariant Decomposer Traits—Model I), (ii) flexible CUE and time-invariant decomposer elemental composition (model II, Section Variable Decomposer Traits—Models II and III), and (iii) flexible CUE and decomposer elemental composition (model III, Section Variable Decomposer Traits—Models II and III). The fitting procedure and the datasets used are described in Section Data Analysis and Model Parameterization. Symbols are defined in Table 1.

**Table 1:** **Definition of symbols and units**.

**Symbol**	**Description**	**Units**
*a, b*	Fitting parameters (Table 3)	Multiple
*C, C*_0_	Litter C content, initial litter C content	gC
(*C*:*N*)_0_	Initial litter C:N ratio	gC gN^−1^
(*C*:*N*)_*B*_	Decomposer biomass C:N ratio	gC gN^−1^
*D*	Decomposition rate	gC y^−1^
*e*	C-use efficiency (growth rate over uptake rate)	–
*e*_0_	Initial C-use efficiency (at *x* = 1; Equation 9)	–
*N, N*_0_	Litter N content, initial litter N content	gN
*p, q*	Auxiliary functions (Equation 5)	–
*r*_0_	Initial litter N:C ratio ($r_{0}={\left(C:N\right)}_{0}^{-1}$)	gN gC^−1^
*r*_*B*_	Decomposer biomass N:C ratio ($r_{B}={\left(C:N\right)}_{B}^{-1}$)	gN gC^−1^
*r*_*B*,0_	Initial decomposer N:C ratio (at *x* = 1; Equation 10)	gN gC^−1^
*t*	Time	y
*u*	Dummy variable of integration (Equation 5)	–
*x*	Fraction of remaining C, *x* = *C*/*C*_0_	–
*y*	Fraction of remaining N, *y* = *N*/*N*_0_	–
α	Coefficient for preferential N uptake	–
β	Slope of *r*_*B*_ (*x*) relation (Equation 10)	gN gC^−1^
ε	Slope of *e* (*x*) relation (Equation 9)	–
ν	Parameter group, ν = α/(1 − *e*_0_ + ε) (Equations 11, 12)	–
ξ	Parameter group, ξ = ε/(1 − *e*_0_ + ε) (Equations 11, 12)	–

### Theory

A cohort of organic matter constitutes the modeled system and is followed during decomposition between an initial state (indicated by subscripts “0”) to a generic time *t*. The system is assumed to be open to exchanges of mineralized products, but closed to new inputs of organic matter. As described in Section Data Analysis and Model Parameterization, the first phase of decomposition, dominated by leaching of soluble organic C and nutrients is removed from the data sets, so that leaching can be neglected in the model as well. Even though this mathematical approach can be applied with some modifications to any nutrient associated to organic matter, here the focus is on nitrogen. Mass balance equations can then be written to describe the temporal evolution of total organic carbon [*C* (*t*)] and nitrogen [*N* (*t*)] in the organic matter cohort, for a given set of initial conditions and decomposer traits (Manzoni et al., 2010),

(1) $$\frac{dC\left(t\right)}{dt}=-D\left[1-e\left(\frac{C}{C_{0}}\right)\right],$$

(2) $$\frac{dN\left(t\right)}{dt}=-D\left[\alpha \frac{N}{C}-e\left(\frac{C}{C_{0}}\right)r_{B}\left(\frac{C}{C_{0}}\right)\right],$$

where *D* is the decomposition rate in C units, *e* is the decomposer community carbon-use efficiency, *r*_*B*_ is the N:C ratio of the decomposer biomass, and α is a coefficient taking into account the chemical heterogeneity of the substrate (α > 1 indicates preferential assimilation of compounds richer in N than the bulk organic matter). The two parameters representing microbial traits (*e* and *r*_*B*_) are written as generic functions of the degree of decomposition, expressed in terms of the fraction of remaining C instead of time (*C*/*C*_0_, where subscript “0” indicates the initial mass of C). It is important to emphasize that as time progresses, *C*/*C*_0_ decreases from 1 to 0; that is, time and *C*/*C*_0_ change in opposite directions during decomposition. Linking traits to *C*/*C*_0_ allows comparing litter decomposition across climatic gradients that affect the degradation rates, but not the underlying relations between microbial community and litter amounts and quality.

With the above assumptions, the only loss in the C balance (Equation 1) is due to decomposer respiration, modeled as the product of the decomposition rate and the fraction of assimilated C not used for growth (i.e., 1 − *e*). The N balance (Equation 2) accounts for the net exchanges of N between decomposers and the inorganic N pool, given by the difference between N supply from organic matter (i.e., $D\alpha \frac{N}{C}$) and the decomposer stoichiometric demand (*Der*_*B*_); the minus sign indicates that N in excess of demand is released (so that $\frac{dN\left(t\right)}{dt}<0$), whereas in case of N shortage, inorganic N is immobilized ($\frac{dN\left(t\right)}{dt}>0$). Similar stoichiometric arguments have already been presented in the context of decomposition in both terrestrial and aquatic systems (Bosatta and Berendse, 1984; Goldman et al., 1987). These equations have the same form as in previous contributions (Manzoni et al., 2010), except for the explicit dependence of decomposer traits (*e* and *r*_*B*_) on remaining C (which introduces nonlinearities in the system of equations) and a slight notation simplification (N:C ratios are used instead of C:N ratios, allowing more compact equations).

It is important to emphasize that Equation (2) is exact only when *r*_*B*_ is time-invariant, but it is an accurate approximation of the exact mass balance as long as $\left|\frac{dr_{B}}{dt}C_{B}|\ll |r_{B}D\right|$. Here, a dependence of *r*_*B*_ on the decomposition state (and thus time) will also be considered. However, at the annual time scale at which the model is interpreted, these changes in *r*_*B*_ are small compared to the decomposition rate *D*, supporting the use of Equation (2) in the following derivations. Moreover, possible climatic effects on *r*_*B*_ are neglected, assuming that inter-annual variations in temperature and water availability have small effect on the mean annual *r*_*B*_ values.

Dividing Equation (2) by Equation (1), a single equation linking organic matter N to organic matter C (instead of *t*) is readily found,

(3) $$\frac{dN}{dC}=\frac{\alpha \frac{N}{C}-\text{e}\left(\frac{C}{C_{0}}\right)r_{B}\left(\frac{C}{C_{0}}\right)}{1-e\left(\frac{C}{C_{0}}\right)}.$$

Defining for convenience the fraction of remaining carbon, *x* = *C*/*C*_0_, the fraction of remaining nitrogen, *y* = *N*/*N*_0_, and the initial litter N:C ratio, *r*_0_ = *N*_0_/*C*_0_, Equation (3) becomes,

(4) $$\frac{dy}{dx}=\frac{\alpha yr_{0}-xe\left(x\right)r_{B}\left(x\right)}{xr_{0}\left[1-e\left(x\right)\right]}.$$

Equation (4) is a linear in *y*, non-autonomous ordinary differential equation that can be solved for the initial condition *y* (*x* = 1) = 1 using the integrating factor method (Boyce and DiPrima, 2009),

(5) $$\begin{array}{l} y\left(x\right)=e^{-p\left(x\right)}\int e^{p\left(x\right)}q\left(u\right)du,\\ p\left(x\right)=-\int \frac{\alpha }{x\left[1-e\left(x\right)\right]}dx,\\ q\left(x\right)=-\frac{e\left(x\right)r_{B}\left(x\right)}{r_{0}\left[1-e\left(x\right)\right]}, \end{array}$$

where *u* is a dummy variable of integration. The analytical solution of Equation (5) requires specific assumptions on the form of *e* (*x*) and *r*_*B*_ (*x*). In the following, the simplest case of time-invariant decomposer traits is considered first, as it allows for an illustrative derivation (Section Time-Invariant Decomposer Traits—Model I). Other cases are discussed in Section Variable Decomposer Traits—Models II and III, and all models are summarized in Table 2. The effects of different choices of *e* (*x*) on the temporal trajectories of litter C are illustrated in Figure S1.

**Table 2:** **Relations between C-use efficiency and fraction of remaining C, ***e*** (***x***), for different assumptions on microbial biomass N:C ratio, ***r***_***B***_ (***x***), and references to the corresponding N release curves, ***y*** (***x***)**.

**Model**	***e* (*x*)**	***r*_*B*_ (*x*)**	***y* (*x*)**	**% with highest *R*^2^**	**% with lowest AIC**
I	*e* = constant	*r*_*B*_ = constant	Equation 6	0	56.1
II	*e*_0_ − ε(1 − *x*)	*r*_*B*_ = constant	Equation 12	70.7	24.4
III	*e*_0_ − ε(1 − *x*)	*r*_*B*,0_ − β(1 − *x*)	Equation 11	29.3	19.5

*The right columns indicate the percentages of datasets in which each model had the highest fraction of explained variance, R^2^, or the lowest Akaike information criterion score (AIC)*.

#### Time-invariant decomposer traits—model I

In the simplest case, CUE and decomposer N:C ratio are assumed to be time-invariant (i.e., *e* (*x*) = *e* and *r*_*B*_ (*x*) = *r*_*B*_; referred to as model I), although both traits could vary across litter types. This assumption has been the basis of previous derivations of analytical nutrient release curves (Bosatta and Staaf, 1982;

Bosatta and Ågren, 1985; Manzoni et al., 2008a, 2010; Ågren et al., 2013). With these assumptions, *p*(*x*) = −αln(*x*)/(1−*e*), and after accounting for the initial condition *y* (*x* = 1) = 1, the fraction of remaining nitrogen is found as (model I),

(6) $$y\left(x\right)=x\frac{e}{\alpha -1+e}\frac{r_{B}}{r_{0}}+\left(1-\frac{e}{\alpha -1+e}\frac{r_{B}}{r_{0}}\right)x^{\frac{\alpha }{1-e}},$$

recovering the equation derived by Manzoni et al. (2010). When α = 1 (bulk litter N:C is representative of the N:C of microbial substrates), the earlier results cited above are also recovered,

(7) $$y\left(x\right)=x\frac{r_{B}}{r_{0}}+\left(1-\frac{r_{B}}{r_{0}}\right)x^{\frac{1}{1-e}}.$$

Because 0 < *e* < 1, the exponent of the second term in Equations (6, 7) is larger than one, so that the whole second term vanishes faster than the first toward the end of the decomposition process (i.e., in the limit for *x* → 0). As a result, the asymptotic litter N:C ratio is given by,

(8) $$\frac{N}{C}\underset{x\to 0}{\to }\frac{e}{\alpha -1+e}r_{B}\approx r_{B},$$

where the last approximation holds as long as α ≈ 1. Equation (8) implies that litter N:C ratio converges toward the microbial biomass N:C ratio as microbial turnover products become an increasingly larger litter fraction. When N is preferentially used, however, α > 1 and hence litter N:C remains lower than the microbial biomass N:C.

#### Variable decomposer traits—models II and III

For generic *e* (*x*) and *r*_*B*_ (*x*) relations, the analytical integration of Equation (5) becomes unfeasible. However, solutions can be found for various functional relations between microbial traits and the fraction of remaining C. For simplicity, linear functions are assumed here,

(9) $$e\left(x\right)=e_{0}-\varepsilon \left(1-x\right),$$

(10) $$r_{B}\left(x\right)=r_{B,0}-\beta \left(1-x\right),$$

where *e*_0_ and *r*_*B*,0_ are the initial CUE and microbial biomass N:C ratio (at *x* = 1), and ε and β are the slopes of the linear relations. The signs of the slopes are not imposed a priori, so ε and β could be either positive (*e* and *r*_*B*_ decrease during decomposition, as *x* decreases) or negative (*e* and *r*_*B*_ increase) depending on the data. The parameterization assuming β = 0 (i.e., time-invariant *r*_*B*_ but flexible *e*) is referred to as model II, while that accounting also for changes in *r*_*B*_ is referred to as model III (Table 2).

With these linear relations and using Equation (5), the fraction of remaining nitrogen is obtained as (model III),

(11) $$\begin{array}{cc} y(x)=x^{\nu }{\left(\frac{1-e_{0}}{1-e_{0}+\varepsilon (1-x)}\right)}^{\nu } & \begin{array}{l} \left\{1+\frac{\varepsilon^{\nu -2}}{2r_{0}{\left(1-e_{0}\right)}^{\nu }}[((1-e_{0}+\varepsilon )(\alpha -2)\beta +(2(r_{\text{B},0}-\beta )+\alpha \beta )\varepsilon ){\text{B}}_{\xi }(1-\nu ,\nu \right)\\ -((1-e_{0}+\varepsilon )(\alpha -2)\beta +(2(r_{\text{B},0}-\beta )+\alpha \beta x)\varepsilon ){\text{B}}_{x\xi }(1-\nu ,\nu )\\ -(1-e_{0}+\varepsilon )(((\alpha -2)\beta +(2(r_{\text{B},0}-\beta )+\beta )\varepsilon ){\text{B}}_{\xi }(1-\nu ,1+\nu )\\ -((\alpha -2)\beta +\;(2(r_{\text{B},0}-\beta )+\beta x)\varepsilon ){\text{B}}_{x\xi }(1-\nu ,1+\nu ))]\}\;, \end{array} \end{array}$$

where $\nu =\frac{\alpha }{1-e_{0}+\varepsilon }$, $\xi =\frac{\varepsilon }{1-e_{0}+\varepsilon }$, and B_*z*_ (*a, b*) is the incomplete Beta function of the variable *z*, with parameters *a* and *b* (Weisstein, 2016).

When the microbial biomass N:C ratio is set to a time-invariant value (i.e., β = 0), Equation (11) simplifies to (model II),

(12) $$\begin{array}{l} y\left(x\right)=x^{\nu }{\left(\frac{1-e_{0}}{1-e_{0}+\varepsilon \left(1-x\right)}\right)}^{\nu }\\ \;\{1+\frac{r_{B,0}}{r_{0}}\frac{\varepsilon^{\nu -1}}{{\left(1-e_{0}\right)}^{\nu }}[{\text{B}}_{\xi }\left(1-\nu ,\nu \right)\\ \;-{\text{B}}_{x\xi }\left(1-\nu ,\nu \right)-\left(1-e_{0}+\varepsilon \right)\\ \;\left({\text{B}}_{\xi }\left(1-\nu ,1+\nu \right)-{\text{B}}_{x\xi }\left(1-\nu ,1+\nu \right)\right)]\}. \end{array}$$

Finally, when also the microbial CUE is time-invariant (i.e., ε = 0), Equation (6) is recovered. Equations (6, 7, 11, and 12) thus represent a hierarchy of models of increasing complexity. In the following, only the latter three equations will be considered, as α is in general higher than one.

### Data analysis and model parameterization

Litterbag decomposition datasets reporting C and N mass decay over time were collected from the literature and online databases. For each litter type and field incubation site, data were further screened to select datasets representative of the whole decomposition process, and ensure a meaningful fitting of the theoretical *y* (*x*) curves. To this aim, datasets in which the fractions of remaining C and N reached values lower than 0.2 and 0.4, respectively, were selected. Moreover, to avoid the risk of overfitting, only datasets with more than eight measurement points were retained. These criteria are more restrictive compared to those adopted in previous studies—a choice justified by the higher flexibility of the *y* (*x*) curves derived here. This selection resulted in 41 datasets including litter from angiosperm grasses, angiosperm trees, and conifer trees, with initial C:N ratios ranging from 15 to 125; i.e., *r*_0_ ranging from 0.008 to 0.067 (Table S1). Litter samples were incubated in field conditions in a range of ecosystems, with mean annual temperature (MAT) ranging from 6 to 28°C, and mean annual precipitation from 300 to 4,000 mm/year (top right panel of Figure S2).

Because the model presented in Section Theory focuses on biological processes and neglects leaching of organic C and N, the initial leaching phase was removed from each dataset (as in Manzoni et al., 2010). Measurement points during the initial leaching phase were identified as those with larger N losses than C losses and simply removed. The initial C and N amounts were updated accordingly. Based on this criterion, significant initial leaching was detected in three out of 41 datasets. It should be emphasized that the stoichiometric model presented here is meant to assess decomposer traits, not predict the rates of litter mass loss, for which a detailed accounting of leaching would be necessary. A single data point characterized by an unrealistically high N concentration (N:C > 0.2) was also excluded, as deemed contaminated.

Litter C and N amounts were normalized by their initial condition, to obtain fractions of remaining C and N to be used for fitting the *y* (*x*) curves. The same normalization was conducted for datasets in which the initial leaching phase had been removed, by recalculating the fractions of remaining C and N based on the litter C and N contents after the end of the leaching phase. The nonlinear least square fitting was conducted in Mathematica environment, using the function NonlinearModelFit (Wolfram Mathematica version 10.0.0.0). Only parameter *e* was obtained by fitting *y* (*x*) curves for model I (Equation 6), whereas both *e*_0_ and ε were obtained by fitting *y* (*x*) curves for models II and III (Equations 11, 12). Values of *e*_0_ and ε were further constrained so that 0 < *e* < 1. Goodness of fit was evaluated by the coefficient of determination and by the finite sample-corrected Akaike information criterion (AIC) scores, which account for both the goodness of fit and the number of fitting parameters (Burnham and Anderson, 2002). The best fitting model has the highest coefficient of determination, while the model that best balances performance and simplicity has the lowest AIC score.

Three parameters were not obtained via nonlinear fitting: the coefficient representing preferential N uptake, α, and the parameters of the *r*_*B*_ (*x*) relation, β and *r*_*B*,0_. The former parameter was set to α = 1.25, based on the observation that the long-term litter N:C ratio is smaller than the microbial biomass N:C, thus requiring α > 1 [Section Time-Invariant Decomposer Traits—Model I; see details in Manzoni et al. (2010)]. The parameters of the *r*_*B*_ (*x*) relation were estimated from measured microbial biomass N:C ratios in decomposing litter. In models I and II, β = 0 and *r*_*B*_ was assumed equal to the long-term average litter microbial N:C ratio of 0.1 [i.e., (*C*:*N*)_*B*_ ≅ 10]. In model III, *r*_*B*_ was assumed to change linearly from the minimum value *r*_*B*,0_ = 0.083 at *x* = 1 (i.e., (*C*:*N*)_*B*_ = 12 at the beginning of decomposition) to the maximum value *r*_*B*_ = 0.125 at *x* = 0 (i.e., (*C*:*N*)_*B*_ = 8 at the end of decomposition). With these values at the beginning and at the end of the decomposition process, the slope is found as β = −0.04. This trend implies an increasing microbial biomass N:C as decomposition progresses and litter N:C ratio increases, as suggested by data (Wagener and Schimel, 1998; van Meeteren et al., 2008; Brandstäetter et al., 2013; Toberman et al., 2014). Moreover, with this parameterization of model III, the value of *r*_*B*_ at *x* = 0.5 is consistent with the long-term average *r*_*B*_ = 0.1 assumed for models I and II.

To assess if the obtained C-use efficiency values are reasonable (although without a rigorous validation), literature data were collected on fungal decomposer C-use efficiency estimates from field and laboratory studies. Four studies specifically investigating litter decomposition in either terrestrial or aquatic systems were found (Frankland et al., 1978; Kominkova et al., 2000; Boberg et al., 2014; Lashermes et al., 2016); the data retrieved from these studies are reported in Table S2.

## Results

The role of decomposer traits and initial litter N availability on the N release curves is illustrated first (Section Effects of Decomposer Traits on the N Release Curves). Second, C-use efficiency and related parameters are estimated for the selected litter decomposition datasets (Section C-Use Efficiency Estimates from N Release Data). Finally, patterns in the estimated traits as a function of stoichiometric and climatic factors are assessed and results from the different C-use efficiency models are compared (Sections Relation between C-Use Efficiency and Litter Stoichiometry and Relation between C-Use Efficiency and Climate). In most analyses, N:C ratios are reported, except when linking C-use efficiency estimates and initial litter stoichiometry, where C:N ratios are used to facilitate comparison with earlier works.

### Effects of decomposer traits on the N release curves

**Figure 1:**
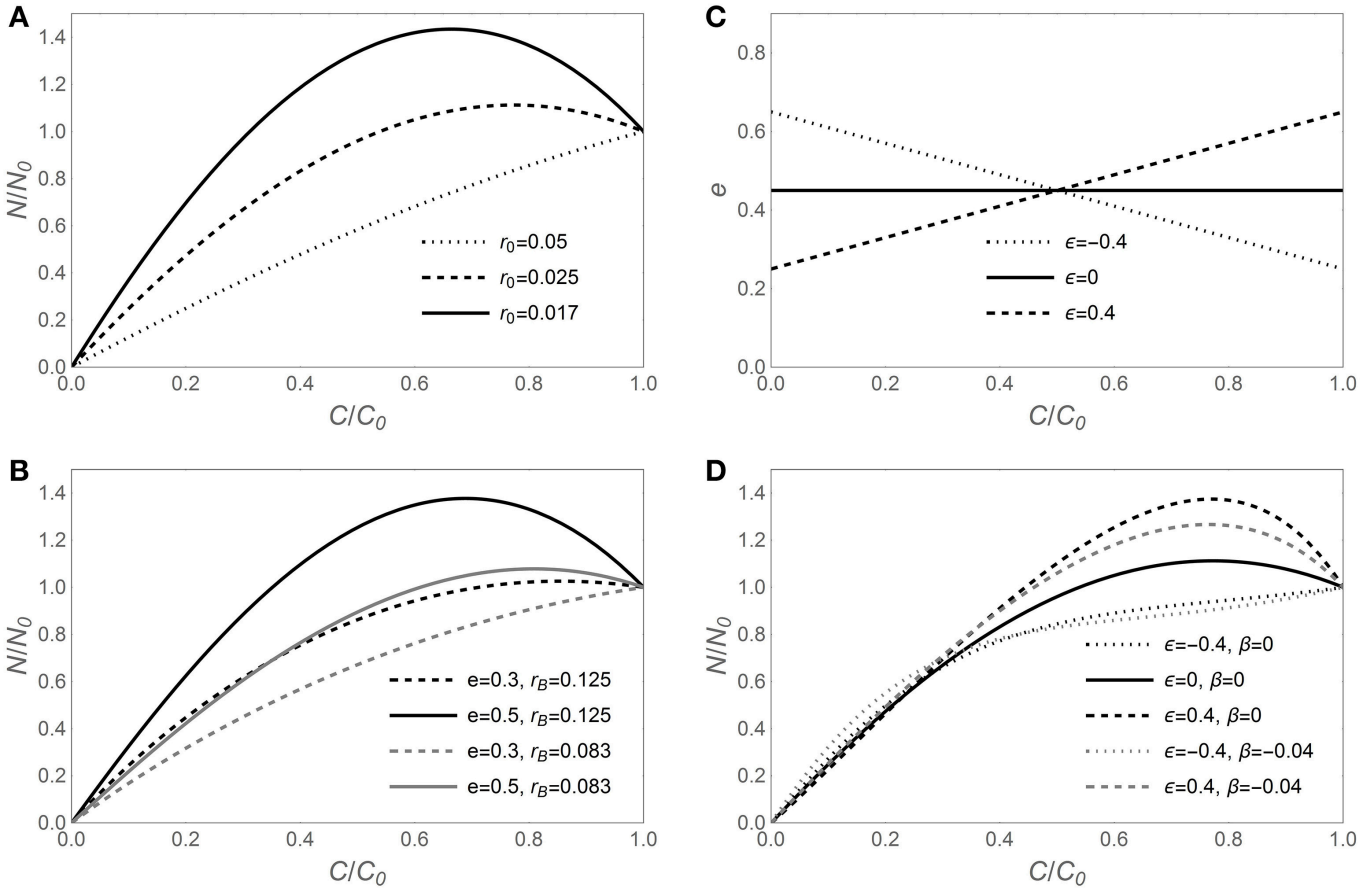
**Effect of contrasting C-use efficiency parameterizations on modeled nitrogen release from decomposing litter, expressed by the fraction of remaining nitrogen (***y*** = ***N***/***N***_**0**_) as a function of the fraction of remaining carbon (***x*** = ***C***/***C***_**0**_)**. Left panels show the predicted *y* (*x*) curves for time-invariant decomposer traits (model I, Equation 6) when varying **(A)** litter N:C ratio (*r*_0_), or **(B)** C-use efficiency (*e*) and decomposer biomass N:C ratio (*r*_*B*_). **(C)** Assumed *e* (*c*) relations (Equation 9) and **(D)** corresponding *y* (*x*) curves when *r*_*B*_ is either time-invariant [β = 0 in Equation (10); *y* (*x*) from Equation (12); model II] or decreasing during decomposition [β = −0.04 in Equation (12); *y* (*x*) from Equation (11); model III]. The same dashing styles are applied in **(C,D)** for the same C-use efficiency model. Unless otherwise specified in the legends of each panel, *e* = 0.45, *r*_*B*_ = 0.1 [(*C*:*N*)_*B*_ = 10], and *r*_0_ = 0.025 [(*C* : *N*)_0_ = 40]; in all panels, α = 1.25. Note that the temporal development in these graphs is from right (*C*/*C*_0_ = 1) to left (*C*/*C*_0_ = 0).

The sensitivity of N release curves to various assumptions on C-use efficiency and microbial biomass C:N ratios is illustrated in Figure 1, starting with the assumption that microbial traits are time-invariant in Figures 1A,B, and then allowing for C-use efficiency and microbial N:C flexibility in Figures 1C,D. In general, lower initial litter N:C ratios require larger N immobilization rates to sustain a homeostatic microbial biomass, resulting in a net increment of N amounts in the litter bags (fraction of remaining N, *y* > 1; Figure 1A). For a given initial litter N:C ratio, microbial stoichiometric traits also affect the shape of the N release curves (Figures 1B–D). Increasing C-use efficiency allows growing more biomass per unit C taken up, but for a given microbial biomass N:C ratio, this translates into higher N requirements and more intense N immobilization (solid vs. dashed curves in Figure 1B). A lower microbial biomass N:C ratio allows growth with lower N supply, because less N is required per unit of C taken up. Hence, N release curves resulting from decomposition by microbial communities with low biomass N:C ratios exhibit a shorter N immobilization phase compared to those resulting from communities with high N:C (gray vs. black curves in Figure 1B).

When the assumptions of time-invariant C-use efficiency and microbial N:C ratio are relaxed, the N release curves do not change their general qualitative behavior, but some quantitative changes emerge (Figures 1C,D). When C-use efficiency increases during decomposition (ε < 0), the N demand in the early decomposition phase is reduced due to relatively low CUE, compared to the case of time-invariant CUE (dotted vs. solid curve in Figure 1D). In contrast, when C-use efficiency decreases during decomposition (ε > 0), the pattern is reversed, and initial N demand is enhanced, resulting in high initial N immobilization (dashed vs. solid curve in Figure 1D). Trends in microbial biomass N:C ratios are compounded with trends in CUE, resulting in less intense N immobilization when microbial N:C is lower in the early decomposition phase (gray vs. black curves in Figure 1D).

### C-use efficiency estimates from N release data

**Figure 2:**
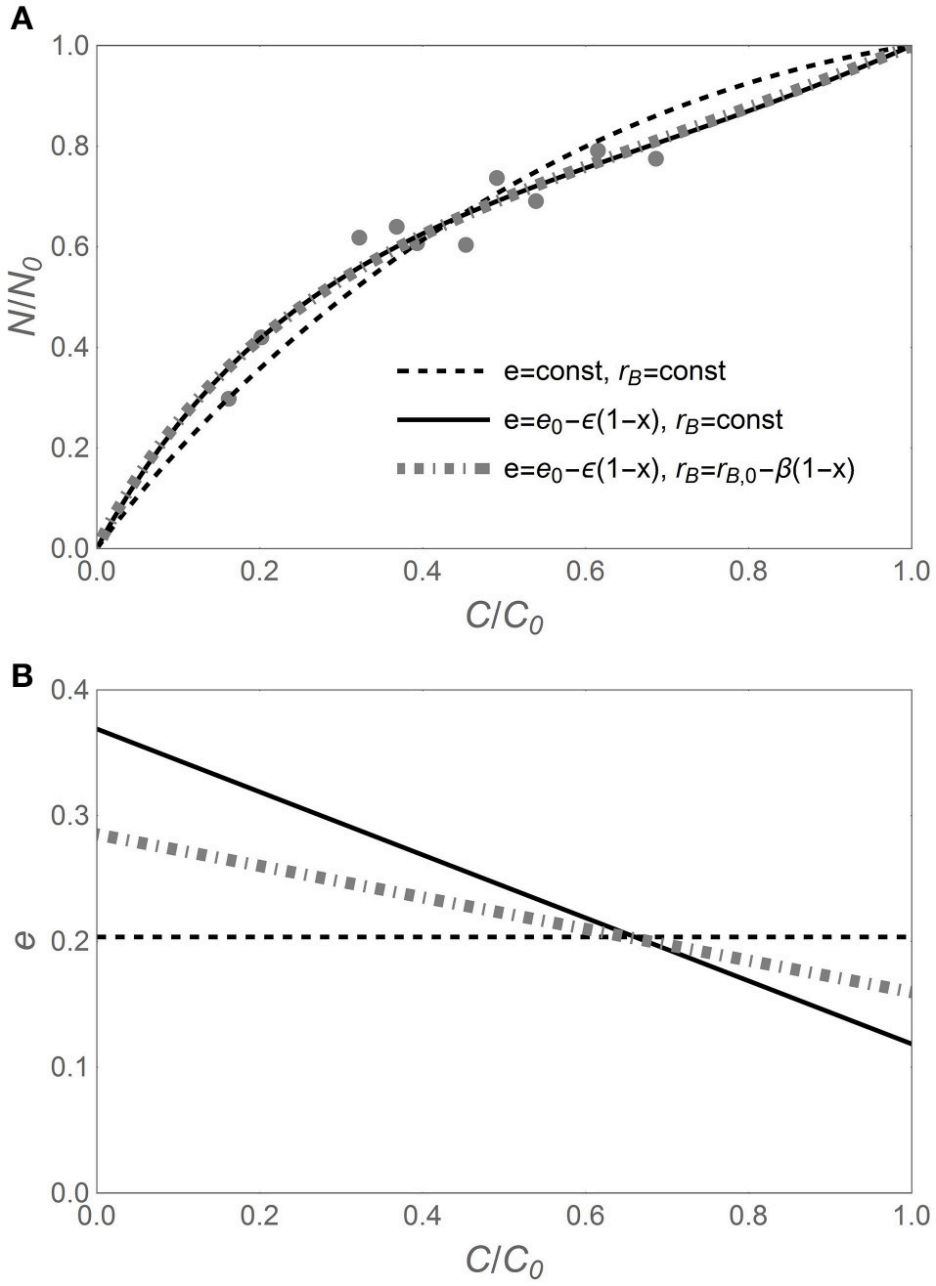
**(A)** Example of nitrogen release trajectory during litter decomposition, expressed as fraction of remaining nitrogen (*y* = *N*/*N*_0_) vs. fraction of remaining carbon (*x* = *C*/*C*_0_). Light gray symbols represent observations (*Macaranga kingii* data from Hirobe et al., 2004), and curves refer to analytical *y* (*x*) relations based on different assumptions on the decomposer traits (see legend). **(B)** Predicted relations between C-use efficiency (*e*) and *x* for the different *y* (*x*) relations. C-use efficiency parameters are obtained via nonlinear least square fitting of the data in A [*e* in Equation (6), and *e*_0_ and ε in Equations (11, 12)]; other parameters: α = 1.25, *r*_*B*,0_ = 0.083 [(*C* : *N*)_*B*_ = 12], and β = −0.04. As in Figure 1, the temporal development is from right (*C*/*C*_0_ = 1) to left (*C*/*C*_0_ = 0).

As suggested by Figure 1, the N release curves based on variable CUE can exhibit a wider range of shapes compared to curves based on time-invariant CUE. This flexibility is required to capture subtle or hidden patterns in the data, as shown in Figure 2. When time-invariant traits are assumed, the best fit *y* (*x*) curve, despite capturing the main trend, overestimates the fraction of remaining N in the early phase of decomposition, while underestimating it in the later phase (black dashed curve). When considering variable CUE (with time-invariant or variable microbial biomass N:C), the model fitting yields ε < 0 (Figure 2B), suggesting that CUE was lower in the early phase than predicted by model I. In turn, lower CUE results in relatively lower N requirements and thus flatter *y* (*x*) (in Figure 2A, compare the solid black and dot-dashed gray curves based on models II and III to the dashed curve of model I). Comparing models II and III, it is evident that trends in microbial N:C ratio partly compensate for trends in CUE, resulting in less negative ε (Figure 2B). In other cases—primarily with N-rich litter—CUE is found to decrease during decomposition (ε > 0), also indicating that the slope parameter ε can be important. Thus, the *y* (*x*) curves based on flexible traits can be more accurate, at the expense of an additional fitting parameter.

**Figure 3:**
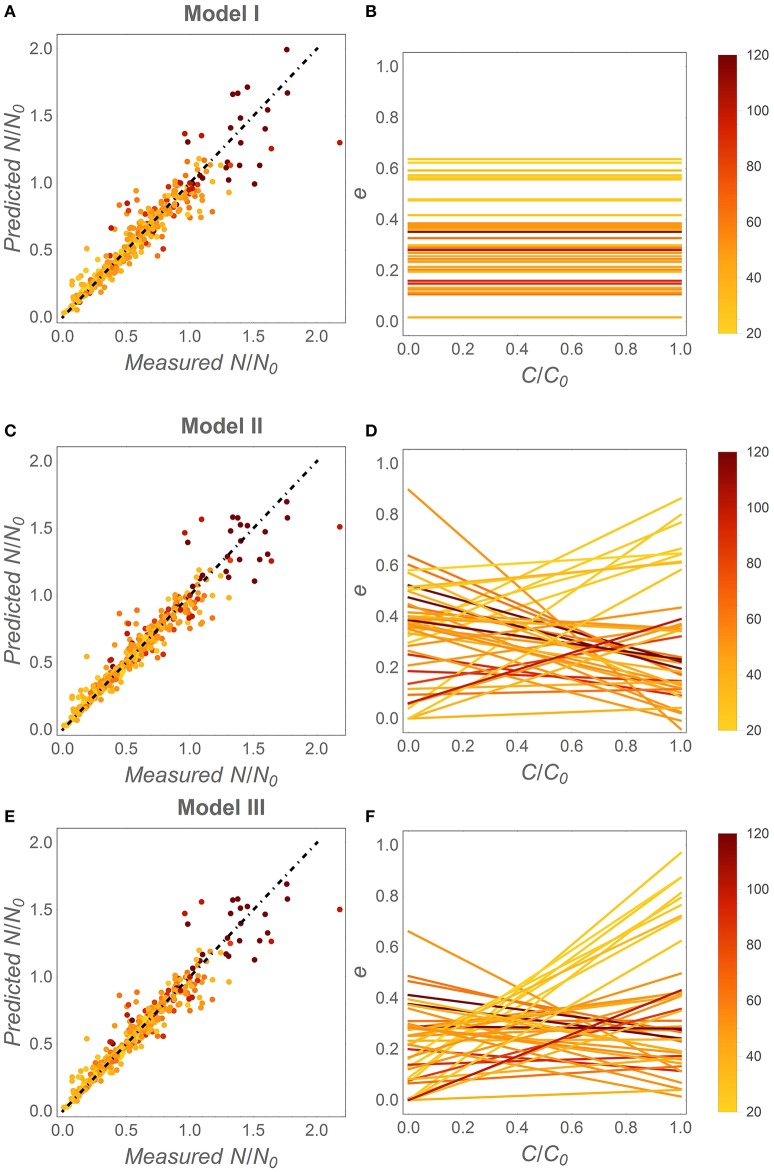
**Fitting results for the different C-use efficiency (***e***) parameterizations**. Left panels: fitting performance by comparing measured and modeled fractions of remaining N (Table 2, Table S1). Right panels: inferred *e* as a function of the fraction of remaining C (*x*). **(A,B)** model I (time-invariant *e* and microbial N:C); **(C,D)** model II [variable *e* (*x*) and time-invariant microbial N:C]; **(E,F)** model III [variable *e* (*x*) and microbial N:C]. Symbols and lines are color coded as a function of the litter initial C:N ratio (color-bars); the dot-dashed lines have unitary slope. Fixed parameters: α = 1.25, *r*_*B*,0_ = 0.083 [(*C* : *N*)_*B*_ = 12], and β = −0.04. As in Figure 1, the temporal development in the right panels is from right (*C*/*C*_0_ = 1) to left (*C*/*C*_0_ = 0).

When fitting the three N release curves to all datasets, some general patterns begin to emerge (Figure 3; all regression results are reported in Table S1). First, all models perform well, with coefficients of determination higher than 0.98 for 75% or more of the datasets (left panels in Figure 3). As expected by the higher number of parameters, models II and III performed marginally better than model I (Table 2). Model II performs better than model III in 70% of the datasets, but differences between the N release curves obtained from the two models are small (as exemplified by Figure 2A). When evaluating model performance by means of AIC scores, model I emerges as the best model in 56% of the cases, followed by model II and III (Table 2). Second, the predicted slopes ε vary between −0.5 and 0.5 when using model II, and between −0.3 and 0.8 when using model III (consistent with the compensation effect of flexible microbial N:C), resulting in a range of *e* (*x*) curves (Figures 3D,F). In general, the slopes ε tend to be negative in most litter types, except for N-rich litter (light-colored lines).

### Relation between C-use efficiency and litter stoichiometry

**Figure 4:**
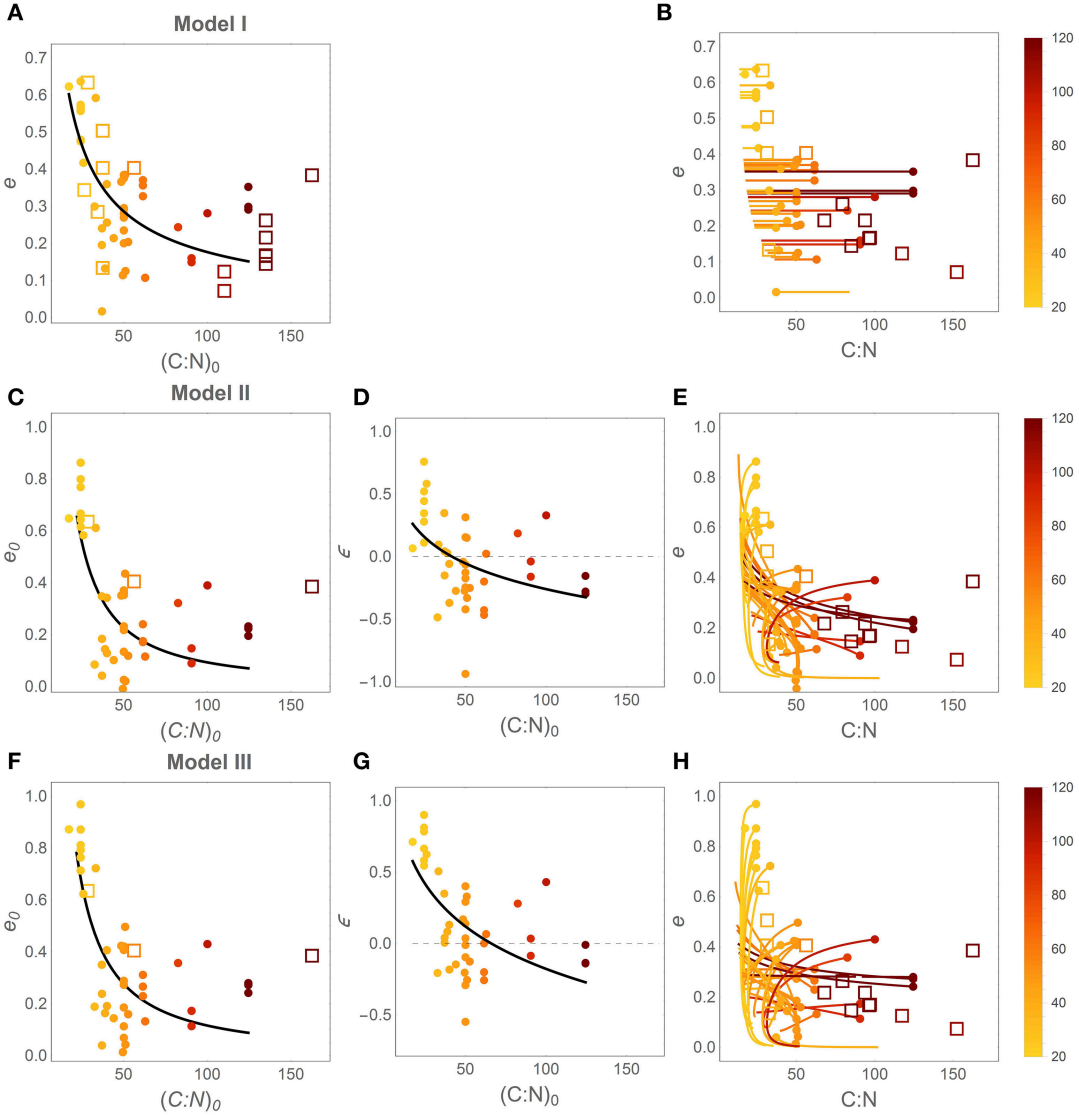
**Effect of litter C:N ratio on C-use efficiency (***e***)**. Left and central panels: relations between initial litter C:N ratio, (*C* : *N*)_0_ = 1/*r*_0_, and the parameters of the different CUE models. Right panels: modeled *e* as a function of litter C:N during decomposition (initial points are identified by filled circles). **(A,B)** model I (time-invariant *e* and microbial N:C); **(C–E)** model II [variable *e* (*x*) and time-invariant microbial N:C]; **(F–H)** model III [variable *e* (*x*) and microbial N:C]. Symbols and lines are color coded as a function of (*C* : *N*)_0_ (color-bars). Closed symbols refer to estimates from model fitting; open symbols refer to observations (Table S2). Regression statistics for the curves in **(A**,**C**,**D**,**F,G)** are reported in Table 3 (all slopes are significantly different from zero). Thin dashed lines in **(D)** and **(G)** indicate ε = 0.

**Figure 5:**
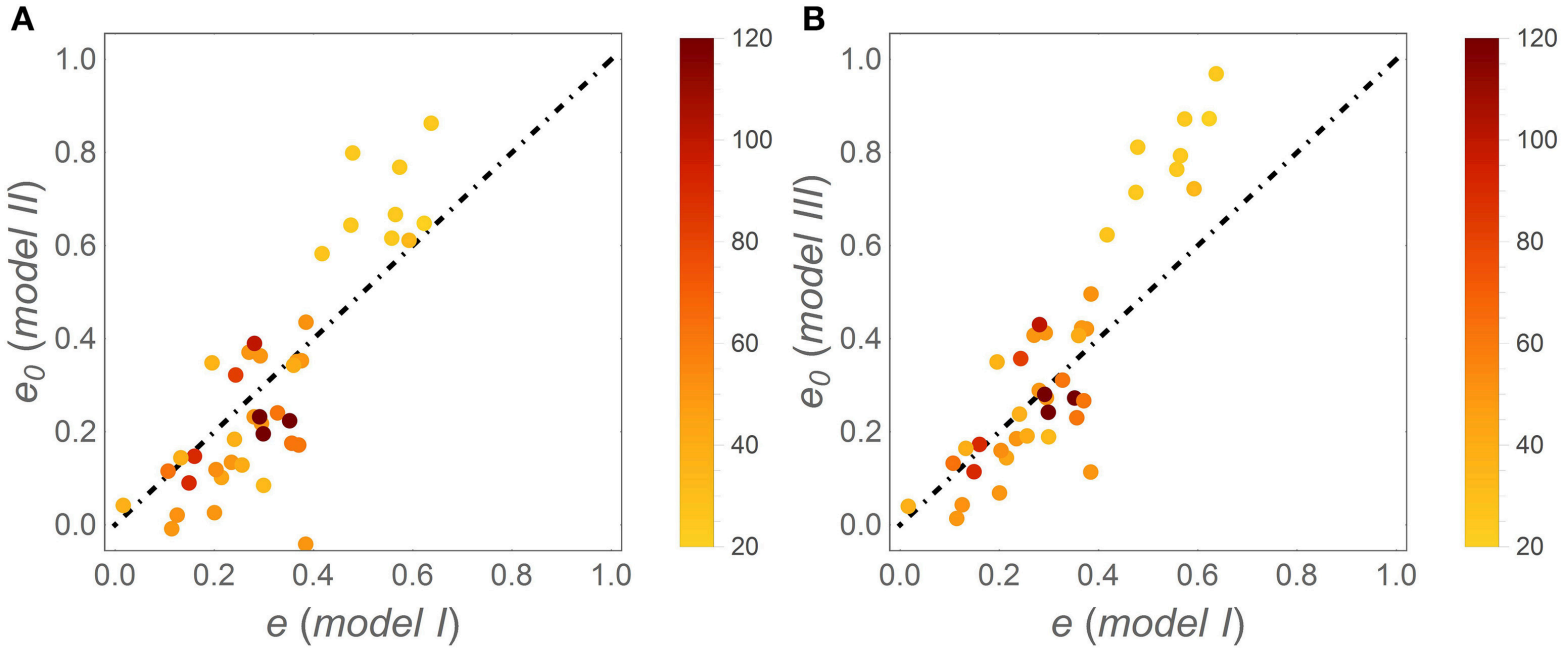
**Correlations between the initial C-use efficiency (***e***_**0**_) estimated using (A)** model II and **(B)** model III, and the time-invariant C-use efficiency estimated from model I (simply denoted as *e*); the dot-dashed lines have unitary slope. Symbols are color coded as a function of (*C* : *N*)_0_ (color-bars).

Patterns in time-invariant *e* (model I) and *e* (*x*) parameters (models II and III) as a function of litter stoichiometry are illustrated in Figure 4. To ease comparisons with previous results, litter C:N ratios are used to characterize litter stoichiometry, instead of N:C ratios. In model I, the estimated *e* strongly decreases with initial litter C:N, (*C* : *N*)_0_ (Figure 4A), consistent with observations (open square symbols). When using models II and III, both the initial CUE (*e*_0_; Figures 4C,F) and the slopes (ε; Figures 4D,G) decrease with increasing (*C* : *N*)_0_ (regression parameters and statistics are reported in Table 3). The initial C-use efficiency for models II and III follows the same pattern as the time-invariant C-use efficiency estimated from model I, as also confirmed by high correlation coefficients between the values of *e*_0_ and those of the time-invariant *e* (Figure 5; correlation coefficients = 0.84 and 0.90, respectively). The three observed initial C-use efficiency values (open square symbols in Figures 4C,F) follow the decreasing trend of the estimated *e*_0_, but tend to be higher in N-poor litter.

**Table 3:** **Relations between C-use efficiency parameters and initial litter C:N ratio, (***C*** : ***N***)_**0**_**.

**Model**	**Fitting function**	***a***	***b***	***R*^2^**
I	$e=a{\left(C:N\right)}_{0}^{b}$	4.4 [0.22, 8.5]	−0.70 [−0.96, −0.43]	0.89
II	$\varepsilon =a\text{ln}\left[\frac{{\left(C:N\right)}_{0}}{b}\right]$	−0.30 [−0.50, −0.094]	42 [28, 56]	0.19
	$e_{0}=a{\left(C:N\right)}_{0}^{b}$	35 [−13, 83]	−1.3 [−1.7, −0.89]	0.81
III	$\varepsilon =a\text{ln}\left[\frac{{\left(C:N\right)}_{0}}{b}\right]$	−0.43 [−0.62, −0.24]	66 [49, 83]	0.44
	$e_{0}=a{\left(C:N\right)}_{0}^{b}$	37 [−6.9, 81]	−1.3 [−1.6, −0.90]	0.85

*Fitting parameters are reported with 95% confidence intervals in square brackets, and fitting performance is quantified by the coefficient of determination, R^2^*.

The slopes of the *e* (*x*) relations are predominantly positive in N-rich litter and negative in N-poor litter (Figures 4D,G), suggesting that in N-rich litter CUE decreases as decomposition progresses, whereas CUE increases in N-poor litter types. These increasing trends of ε with increasing initial litter N concentration are statistically significant for both models, as indicated by *a* < 0 in the ε[(*C* : *N*)_0_] regression models reported in Table 3. The C:N ratio at the transition between positive and negative slopes is given by parameter *b* in the ε[(*C* : *N*)_0_] relations. The C:N ratios at the transition are estimated as 42 and 66 for models II and III, respectively, and both values are significantly higher than zero, confirming that there is a statistically significant shift from increasing to decreasing CUE along a gradient of litter N availability.

Finally, Figures 4B,E,H show the trajectories of CUE and litter C:N as emerging from the modeled *y* (*x*) curves. To draw these curves, C:N ratios are calculated as *x* (*C* : *N*)_0_/*y* (*x*) (these are the actual variable C:N ratios, not the constant initial value), and CUE trends as *e* (*x*). Thus, each curve describes trajectories of CUE and C:N as the fraction of remaining C decreases from 1 to 0. While in Figure 4B CUE remains stable due to the underlying assumption of model I, in Figures 4E,H trajectories are more complex. The dark and orange curves referring to litter with initial C:N ratio above ~50, exhibit increasing CUE as litter C:N decreases (N:C increases) during decomposition. In contrast, light colored, “C-shaped” curves corresponding to N-rich litter exhibit decreasing CUE as the C:N ratio initially decreases, but then mildly increases toward the final phase of decomposition (N:C first increases and then decreases). Observed C-use efficiency values broadly overlap with the estimated *e* (*C* : *N*) trajectories (compare open symbols and solid curves in Figures 4B,E,H).

### Relation between C-use efficiency and climate

In contrast to the clear patterns of CUE and related parameters in relation to litter stoichiometry, mean annual temperature (MAT) and precipitation (MAP) do not appear to have an effect on CUE. The slopes of linear relations between *e* (model I) or *e*_0_ and ε (models II and III), and MAT or MAP are not significantly different from zero, suggesting lack of significant climatic effects (Figure S3). Climatic effects are also weak or inconsistent when considering specific litter types incubated at different sites (results not shown).

## Discussion

### A stoichiometric approach for decomposer trait estimation

The analysis of microbial growth kinetics is based on the idea that microbial traits can be mapped into parameters of process models. These parameters can then be obtained by fitting the model results (either analytical equations or numerical solutions) to observations (Panikov, 1995). While mapping traits into model parameters can be relatively simple in idealized laboratory conditions with minimal microbial and substrate diversity, it can be challenging in complex soil systems, where both traits and model parameters must be interpreted as “macroscopic” properties that integrate the underlying heterogeneities (Manzoni et al., 2008b). Nevertheless, microbial community traits have also been obtained by fitting models to soil incubation studies (Nicolardot et al., 2001; Pansu et al., 2004) and litter decomposition datasets (Wetterstedt and Ågren, 2011; Manzoni et al., 2012a). Here the same conceptual approach is applied, but instead of fitting model results as a function of time, analytical relations between two elements are obtained (Manzoni et al., 2008a; Ågren et al., 2013). This method focuses on stoichiometry rather than kinetics and reduces the degrees of freedom of the problem, thereby allowing more robust trait estimation.

The microbial community C-use efficiency is particularly challenging to measure and interpret (Geyer et al., 2016), motivating the use of the proposed stoichiometric approach as an alternative tool for CUE estimation. CUE has been hypothesized to vary in response to nutrient availability (Sterner and Elser, 2002; Manzoni et al., 2008a), but also microbial elemental composition could change as cells try to compensate nutrient imbalances. Unfortunately, the roles played by decomposer elemental composition and CUE in the nutrient release curves employed here to estimate microbial traits are difficult to disentangle. In fact, either decreasing microbial biomass N:C or decreasing *e* reduces the nutrient demand per unit C consumed (as demonstrated analytically by Ågren et al., 2013, and as illustrated in Figure 1). In two studies where both parameters were fitted via nonlinear regression (Ågren et al., 2001; Nicolardot et al., 2001), contrasting results were found. In one case, microbial N:C increased significantly with amendment N:C ratio, while *e* tended to reach the upper bound set as a constraint for parameter optimization (Nicolardot et al., 2001). In the other case, microbial N:C remained stable, while *e* increased with increasing inorganic nutrient availability and litter N:C ratio (Ågren et al., 2001). These opposite results confirm the covariation of *e* and *r*_*B*_ and suggest strong sensitivity to the fitting procedure, unless some additional constraints are imposed. This issue has been dealt with in different ways: by imposing a time-invariant *r*_*B*_ and letting *e* vary (Manzoni et al., 2008a, 2010), or by estimating the product *er*_*B*_ (corresponding to the N:C ratio at incipient N immobilization) without distinguishing between the two parameters (Ågren et al., 2013). Because *r*_*B*_ values are relatively more constrained than CUE values, here a time-invariant *r*_*B*_ (models I and II) or a pre-defined relation between *r*_*B*_ and the fraction of remaining C (model III) is assumed. Comparing models II and III suggests little impact of *r*_*B*_ trends on CUE, as also discussed in Section Stoichiometric Drivers of Decomposer Trait Flexibility.

Two other confounding factors could complicate the interpretation of the results: leaching and variations in the coefficient α. The former could contribute to losses of organic C and N from the litter system independently of microbial traits, thereby affecting the estimated CUE. Because the proposed modeling framework focuses on microbial-driven decomposition and cannot capture decomposition trajectories when physical processes dominate, the initial leaching phase was removed when evident in the data. This procedure only affected three out of 41 datasets, suggesting that in the selected decomposition trajectories initial leaching was not as important as microbial processes. Besides the initial leaching, it is possible that residual leaching losses occurred in wet sites throughout the decomposition process. This residual leaching (referred to simply as “leaching” in the following) had been included in a previous work employing a comparable model (Manzoni et al., 2010), but was neglected here for simplicity. An increase in leaching rate would increase the estimated CUE, because losses of organic C via leaching occur “in parallel” with respiration. Thus, for a given (measured) C loss, assuming larger leaching implies lowering the contribution of respiration, which translates into higher CUE estimates. Therefore, if leaching was higher in the early decomposition phase, the initial CUE was overestimated. As a consequence, the actual CUE trends should be weaker when the slope ε > 0, but stronger when ε < 0, because initial CUE was overestimated. However, mean annual precipitation, which could be expected to be correlated to leaching rates, has no significant effect on any of the CUE parameters (Figure S3), suggesting that CUE estimates are not significantly biased by neglecting leaching.

Increasing decomposer preference for N (higher α), by improving organic N availability, reduces N immobilization (Figure S2). Hence, for a given (measured) N release curve, higher α results in higher estimated CUE. However, it is not clear how α could change during decomposition. Leachate elemental ratios, which could be hypothesized to be more representative of microbial substrate than the bulk litter ratios, are variable (Magill and Aber, 2000; Michalzik et al., 2001; Lashermes et al., 2016) and in some cases leachate N:C ratios are even lower than in the bulk litter (Fanin et al., 2013). Without more specific information on temporal trends in α, the constant value α = 1.25 appears to be a reasonable choice. As an alternative, more complex models including a dissolved organic matter pool could be useful. However, increased model complexity would also introduce further uncertainties.

Despite difficulties in isolating the effects of CUE from those of other traits, the proposed method (i) provides a highly accurate description of N release trajectories (Table 2), and (ii) identifies trait patterns that are consistent with current conceptual understanding of decomposition (as discussed in Section Stoichiometric Drivers of Decomposer Trait Flexibility). Empirical quantification of microbial community CUE and elemental composition in long-term litter incubations could help validate this approach and disentangle the effects of possible confounding factors.

### Stoichiometric drivers of decomposer trait flexibility

Microbial traits associated with metabolic processes often vary along gradients of nutrient availability in both microbial isolates and communities. This trait flexibility at the community level is required to reduce imbalances in nutrient supply (e.g., by tuning extracellular enzyme expression; Sinsabaugh et al., 2014), and to compensate stoichiometric imbalances by changing body composition (Tezuka, 1990; Godwin and Cotner, 2015), altering metabolism (Sterner and Elser, 2002; Manzoni et al., 2008a), or shifting community composition (Cotner et al., 2010; Godwin and Cotner, 2014). While trait variations have been mainly studied across nutrient availability treatments, temporal trends in traits along nutrient availability trajectories are less clear. In this context, litter decomposition represents a useful model system thanks to the wide range of nutrient conditions during degradation of a single cohort and the abundance of data tracking the temporal changes of litter C and nutrient pools.

Even though N:C ratios of individual microbial strains can vary significantly (Mouginot et al., 2014), decomposer communities appear homeostatic with respect to N across wide ranges of organic matter elemental composition (Fanin et al., 2013; Xu et al., 2013). However, in decomposing litter undergoing strong nutrient enrichment, some trends in microbial biomass N:C have been found. In some studies, microbial N:C increases with increasing litter N:C as decomposition progresses (Wagener and Schimel, 1998; van Meeteren et al., 2008; Brandstäetter et al., 2013; Toberman et al., 2014), but in others no trends are apparent (Mooshammer et al., 2014a). Along the extreme stoichiometric gradient between a decaying log and the nearby soil (assuming that the latter is representative of the final phases of wood decomposition), only small differences in microbial biomass elemental composition were found, despite the four-fold increase in N:C ratio (Hart, 1999). Overall, this evidence suggests that N availability during decomposition of a litter cohort might not always be a good predictor of microbial biomass elemental composition. Here, *r*_*B*_ flexibility is accounted for in model III, but does not affect the main patterns predicted for CUE (Figures 3, 4), suggesting that stoichiometric flexibility of litter microbial communities may not be sufficient to compensate strong nutrient imbalances.

The assumed increase from *r*_*B*_ = 0.083 to 0.125 (i.e., (*C* : *N*)_*B*_ decreases from 12 to 8) is consistent with observed changes, even though lower N:C ratios have also been found. Had a steeper decrease in *r*_*B*_ with decreasing fraction of remaining C been considered, stronger differences between results from model II and III would have emerged. However, the lower N:C ratios observed in some litter microbial communities might not be representative of the actively growing (and relatively nutrient-rich) fraction of the microbial community that the stoichiometric model is meant to describe.

If the cellular composition is too stable to compensate stoichiometric imbalances, it can be surmised that metabolic processes might provide the required flexibility (Manzoni et al., 2008a; Mooshammer et al., 2014b). Observed C-use efficiency values support this view by strongly decreasing as the initial litter C:N ratio (Figures 4A,C,F) as well as the instantaneous litter C:N (Figures 4B,E,H) increase across litter types. Evidence from an experiment designed to test this hypothesis is supportive (Lashermes et al., 2016), although the measured CUE values are higher than expected from model results in N-poor litter. In this dataset, however, fungal biomass—and consequently CUE—might have been overestimated (Lashermes et al., 2016), thus explaining the mismatch. The expected decline in CUE at high substrate C:N ratio is not always observed in culture studies, suggesting that some organisms may exhibit sufficient flexibility in their cellular composition to compensate nutrient imbalances and that the effects of compound C- and nutrient-limitation are not easy to predict (Keiblinger et al., 2010).

The temporal trends in C-use efficiency are potentially more complex to interpret than the trends in decomposer biomass elemental composition. If CUE is reduced in response to nutrient limitation, it can be hypothesized that CUE increases during decomposition, as the nutrient availability increases (in this framework, this trend would be characterized by ε < 0). However, such an effect would be apparent only in litter types with high initial C:N ratio. In contrast, as recalcitrant material and microbial by-products accumulate in the late decomposition phases (Berg and McClaugherty, 2003), acquiring C could require larger investments in extracellular enzymes (Ågren and Bosatta, 1987), and microbial populations would spend more time in relatively inactive states associated with higher respiration per unit C taken up and thus lower CUE. Experimental evidence indeed showed that decomposers degrading lignin exhibit lower CUE than communities feeding on higher quality substrates (Bahri et al., 2008). Moreover, increased N concentration in this late phase is often associated with reduced decomposition rates, due to inhibition of extracellular enzymes (Berg and McClaugherty, 2003; Hobbie et al., 2012). As a result, in such conditions CUE could decrease with progressing decomposition (ε > 0). This effect would be apparent only in N-rich litter where C would become limiting. A similar pattern of higher initial CUE in N-rich litter than in N-poor litter, followed by a reversed pattern in the late decay phase had also been hypothesized by Cotrufo et al. (2013). As shown in Figure 4, the patterns in ε estimated with models II and III are in line with these expectations, with positive ε values in N-rich litter types, transitioning to negative ε values at high initial litter C:N ratios.

The initial CUE values of models II and III follow the same decreasing pattern with increasing initial litter C:N as the time-invariant CUE of model I (Figure 5). It can thus be concluded that regardless of the specific time trajectory of CUE, the initial CUE compensates litter stoichiometric imbalances by reducing the decomposer growth rate and nutrient demand in N-poor litter. Moreover, the initial CUE values are representative of the first and most active phase of decomposition, so that the same pattern remains evident when considering the long-term average CUE, as in model I. Hence, this result lends some support to the use of time-invariant CUE to detect stoichiometric effects on CUE across broad litter quality gradients (Manzoni et al., 2008a), although temporal trends are key to understanding the interplay between N- and C-limitation during degradation of a single litter cohort.

### Climatic drivers of decomposer trait flexibility

Mean annual temperature and precipitation explain a large fraction of the observed variability in litter decay constants (Aerts, 1997; Adair et al., 2008). For a given litter type, incubation under conditions ranging from cold or dry to warm and moist causes a more than 10-fold variation in the decay constants (Adair et al., 2008). Such a large climatic effect could be expected to also appear when investigating broad-scale trends in C-use efficiency and related parameters. However, climatic factors do not play any significant role (Figure S3), at least at the scale of this analysis, as previously noted by Manzoni et al. (2008a). Thus, two complementary effects of climate on decomposition are occurring. While warm and moist conditions are favorable for decomposers and thus promote rapid litter decomposition, the way C is partitioned between growth and respiration does not seem to be affected, suggesting a separation between the drivers of microbial metabolic rates (climate) and those affecting metabolic efficiency (litter stoichiometry). Hence, climate is expected to affect C sequestration by altering the balance of inputs to the soil and respiration rates, while litter elemental composition largely affects the patterns of nutrient release and the metabolic efficiency of the decomposers.

Had an increase in leaching rate with mean annual precipitation been accounted for, the CUE estimates would have been higher for the wetter sites, potentially introducing a positive correlation between CUE and precipitation. Dissolved organic C production from litterbags incubated in a lake amounted to about 20% of mass loss (Kominkova et al., 2000). Even assuming that this value is representative for leaching in the wettest terrestrial ecosystems (10% is a more reasonable figure, Michalzik et al., 2001), variability in our CUE estimates would still be driven by litter stoichiometry rather than precipitation.

Both short-term fluctuations in soil moisture (e.g., Tiemann and Billings, 2011) and incubation at different temperatures (e.g., Frey et al., 2013) have been shown to affect CUE of soil microbial communities in laboratory conditions. In particular, CUE tends to decline with increasing temperature, although its temperature sensitivity is still a matter of debate, and when discounting the effect of mortality, stable CUE values have been found (Hagerty et al., 2014). In contrast, positive temperature effects on CUE were found in a global-scale study, in which CUE was estimated using a model driven by the stoichiometric ratios of substrates and ecoenzymatic activities (Sinsabaugh et al., 2016). It is conceivable that when integrating responses to environmental fluctuations and microbial community dynamics at the annual time scale considered here, more stable CUE values than under laboratory conditions are achieved. Furthermore, physiological factors such as temperature acclimation, or shifts in microbial community composition, can contribute to reducing temperature sensitivity in the long-term (Frey et al., 2013; Allison, 2014; Sinsabaugh et al., 2016). These processes could explain why the CUE values of decomposer communities degrading the same litter type along a climatic gradient (datasets from the LIDET study) are weakly or inconsistently dependent on climate, but strongly dependent on litter quality.

### On the interpretation of community-scale C-use efficiency

The estimated CUE values are to be interpreted at the microbial community scale (sensu Geyer et al., 2016) and should be regarded as “effective,” lumped parameters that capture the average behavior of a biologically and chemically heterogeneous system (Manzoni et al., 2008b). In fact, the proposed model integrates the contributions of all decomposer to the bulk community metabolism. However, the CUE estimates presented here are not confounded by microbial turnover, which is implicitly accounted for as a recycling flux into the litter pool. Community dynamics can generate patterns in CUE that differ from those of the individual constituents or a homogeneous community (Kaiser et al., 2014). Results from an individual-based model show that CUE can remain high regardless of litter C:N ratio, because turnover of N-rich microbial products allows at least part of the community to feed on substrates with substantially higher N:C than the bulk litter (Kaiser et al., 2014). This effect could be captured by higher values of α in this framework, but such an adjustment would not be supported by independent information. While considering lumped processes aids in extracting information from relatively coarse data (such as C and N mass in litter samples), it also represents a limitation of this approach, as it precludes the possibility of further disentangling the mechanistic drivers of flexible community-level traits. It would be fruitful to combine litter decay data with detailed community composition and physiological measurements that can assist in interpreting patterns in the estimated community-level traits.

## Conclusions

A stoichiometric model is presented as a tool for quantifying variations in decomposer traits. Specifically, changes in microbial community C-use efficiency across litter types and through time during decomposition of individual litter cohorts are estimated by fitting analytical N release curves to litter C and N mass data. This method offers insights on decomposer traits that would be difficult to measure, and allows generating specific hypotheses that could be targets of more detailed empirical studies. C-use efficiency is found to be flexible, showing a continuum of responses during decomposition. In general, C-use efficiency increases along a gradient of litter types with increasing N availability. Temporal patterns in a single litter cohort are more complex. N-poor litter types tend to exhibit increasing CUE possibly due to large stoichiometric imbalances and N-limitation in the early phase of decomposition, followed by improved N status and consequently more efficient biomass production. In contrast, N-rich litter types exhibit lowering CUE, possibly driven by C-limitation, increased chemical complexity and inhibiting effects of high N availability in the late phase of decomposition. Hence, these data-driven analysis suggests that trajectories of decomposer community traits depend in a strongly nonlinearly way on litter stoichiometry, as the decomposers transition from N- to C-limited conditions.

## Author contributions

SM designed the study, developed the theory, conducted the analyses, and wrote the manuscript.

## Funding

Funding was provided by the Bolin Centre for Climate Research (Research Area 4), through the project “Scaling carbon-use efficiency from the organism- to the global-scale,” and by the Swedish Research Councils, Formas (2015-468), and VR (2016-04146). Significant funding for the LIDET data was provided by the National Science Foundation Long-Term Ecological Research program.

### Conflict of interest statement

The author declares that the research was conducted in the absence of any commercial or financial relationships that could be construed as a potential conflict of interest.
