# Supplementary material for: Flexible Carbon-Use Efficiency across Litter Types and during Decomposition Partly Compensates Nutrient Imbalances—Results from Analytical Stoichiometric Models
Source: Front Microbiol. 2017 Apr 26;8:661. doi: 10.3389/fmicb.2017.00661 (PMC5405148; doi:10.3389/fmicb.2017.00661)
Supplement: Supplementary file 1 [file Table1.pdf]

Supplementary Information

Flexible carbon-use efficiency across litter types and during decomposition partly compensates nutrient imbalances – Results from analytical stoichiometric models

Stefano Manzoni*

***Correspondence**

Stefano Manzoni

stefano.manzoni@natgeo.su.se

Supplementary tables

Table S1. Datasets used and summary of estimated carbon-use efficiency and related parameters.

Species	MAT	MAP	$(C:N)_0$	Model I	Model II		Model III		Source
	(degC)	(mm)		e	e_0	ε	e_0	ε	
Drypetes glauca	10	2500	24.3	0.56	0.67	0.280	0.795	0.665	LIDET dataset (Harmon, 2005)
Pinus resinosa	9	311	90.5	0.15	0.089	-0.161	0.115	-0.085	
Acer saccharum	13	1800	61.5	0.33	0.241	-0.199	0.311	0.001	
Drypetes glauca	13	1800	24.3	0.48	0.646	0.346	0.712	0.582	
Quercus prinus	13	1800	50.0	0.23	0.134	-0.246	0.184	-0.096	
Drypetes glauca	7	1119	24.3	0.64	0.86	0.520	0.97	0.900	
Quercus prinus	7	1119	50.0	0.29	0.216	-0.174	0.27	-0.010	
Acer saccharum	9	920	61.5	0.36	0.172	-0.428	0.234	-0.256	
Quercus prinus	9	920	50.0	0.27	0.374	0.314	0.411	0.401	
Quercus prinus	25	4000	50.0	0.38	0	-0.938	0.112	-0.548	
Triticum aestivum	25	4000	124.5	0.35	0.221	-0.299	0.271	-0.139	
Pinus resinosa	25	2377	90.5	0.16	0.151	-0.039	0.174	0.034	
Acer saccharum	18	2300	61.5	0.37	0.173	-0.467	0.27	-0.200	

Drypetes glauca	18	2300	24.3	0.56	0.611	0.111	0.765	0.545	
Quercus prinus	18	2300	50.0	0.38	0.356	-0.064	0.419	0.139	
Drypetes glauca	10	3200	24.3	0.48	0.799	0.759	0.811	0.811	
Quercus prinus	10	3200	50.0	0.20	0.028	-0.422	0.069	-0.291	
Triticum aestivum	10	3200	124.5	0.30	0.191	-0.279	0.245	-0.135	
Drypetes glauca	21	1350	24.3	0.57	0.764	0.444	0.874	0.784	
Quercus prinus	21	1350	50.0	0.28	0.233	-0.127	0.287	0.037	
Thuja plicata	21	1350	82.4	0.24	0.326	0.186	0.36	0.280	
Triticum aestivum	21	1350	124.5	0.29	0.236	-0.154	0.281	-0.009	
Mallotus japonicus	10	2495	50.0	0.29	0.365	0.155	0.412	0.292	(Osono and Takeda, 2004, 2005)
Carpinus laxiflora	10	2495	48.5	0.37	0.349	-0.041	0.42	0.170	
Swida controversa	10	2495	37.0	0.24	0.18	-0.150	0.236	0.006	
Pterostyrax hispida	10	2495	17.2	0.62	0.646	0.066	0.871	0.711	
Dendrocalamus strictus	NA	936	50.8	0.38	0.43	0.150	0.499	0.329	(Tripathi and Singh, 1992)
Dendrocalamus strictus	NA	936	32.6	0.30	0.084	-0.486	0.184	-0.206	
Grass mix	NA	936	44.0	0.21	0.105	-0.275	0.144	-0.146	
Anacardium occidentale	27.4	1619	33.2	0.59	0.616	0.096	0.726	0.506	(Isaac and Nair, 2005)

Mesua borneensis	26	3850	62.8	0.11	0.113	0.023	0.137	0.067	(Hirobe et al., 2004)
Ficus brunneo-aurata	26	3850	39.9	0.36	0.341	-0.059	0.412	0.132	
Macaranga kingii	26	3850	52.6	0.20	0.12	-0.250	0.165	-0.125	
Oubanguia alata	NA	5393	38.8	0.13	0.148	0.028	0.163	0.083	(Chuyong et al., 2002)
Strephonema pseudocola	NA	5393	49.5	0.11	0	-0.274	0.016	-0.204	
Schima wallichii	21.8	2680	51.0	0.13	0.019	-0.331	0.045	-0.255	(Xu and Hirata, 2005)
Daphniphyllum glaucescens	21.8	2680	40.0	0.26	0.13	-0.370	0.188	-0.182	
Bambusa pervariabilis x Dendrocalamopsis daii	16.2	1490	36.9	0.20	0.348	0.348	0.35	0.350	(Tu et al., 2014)
Eucalyptus grandis	16.2	1490	37.0	0.02	0.042	0.042	0.04	0.040	
Neosinocalamus affinis	16.2	1490	25.9	0.42	0.583	0.583	0.623	0.623	
Pleioblastus amarus	16.2	1490	100.0	0.28	0.39	0.330	0.43	0.430	

NA: not available from the original publications.

Table S2. Measured C-use efficiency of fungal decomposers. All C:N ratios are on a mass basis.

Fungal species or genus	Temperature (degC)	Litter C:N at CUE measurement time	Initial litter C:N	Fungal biomass C:N	CUE	Source
Gymnopus	20	67.9	135	30	0.212	(Boberg et al., 2014)
Gymnopus	20	93.8	135	30	0.212	
Gymnopus	20	79.4	135	30	0.258	
Mycena	20	85.1	135	32	0.141	
Mycena	20	97.1	135	32	0.164	
Mycena	20	96.4	135	32	0.162	
Mycena galopus	15	NA	26.5	12.4	0.340	(Frankland et al., 1978)[1]
Mycena galopus	15	NA	34.4	12.4	0.281	
Phanerochaete chrysosporium	25	28.5	28.5	7.1	0.630	(Lashermes et al., 2016)[2]
Phanerochaete chrysosporium	25	162.6	162.6	7.1	0.380	
Phanerochaete chrysosporium	25	56.2	56.2	7.1	0.400	
Fungal community	7.5	32.5	37.5	NA	0.13	(Kominkova et al., 2000)[3]
Fungal community	7.5	31.4	37.5	NA	0.5	
Fungal community	7.5	31.3	37.5	NA	0.4	

Fungal community	6.9	117.4	110.2	NA	0.12
Fungal community	6.9	152.3	110.2	NA	0.068

NA: not available from the original publications.

1 Only CUE estimated with the hexosamine assay (considered more accurate in the original publication) are used.

2 Due to the short incubation period before CUE estimation (14 days), the litter C:N ratio at the CUE measurement time was assumed equal to the initial litter C:N ratio.

3 Data from the first sampling date are excluded as deemed not representative of long-term decomposition (samples were obtained immediately after bag preparation, without leaving time to the decomposer community to equilibrate). Incubation in shallow water of a freshwater lake.

Supplementary figures

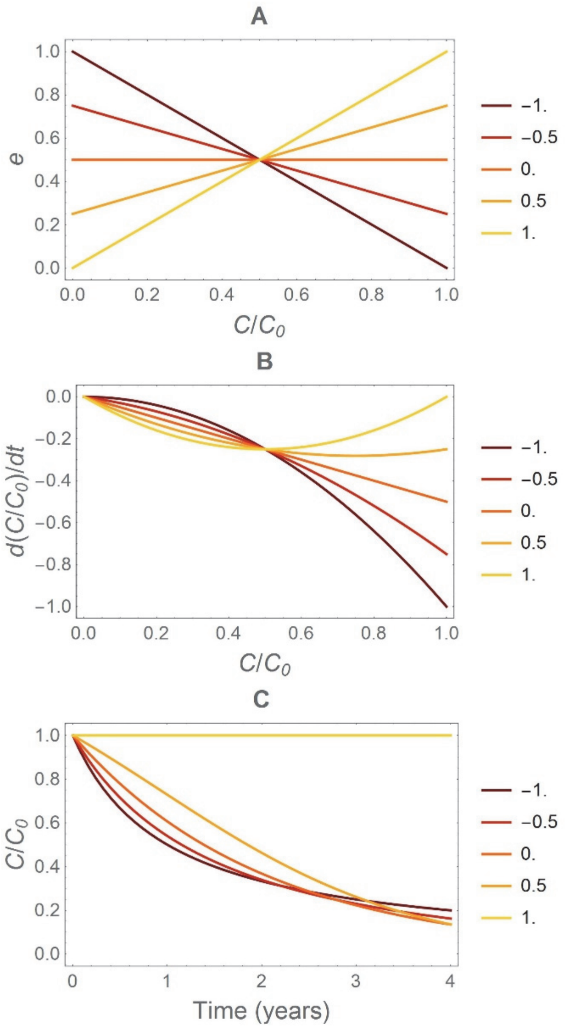

Figure S1. Illustration of the effects of variable C-use efficiency (e) on the temporal trajectories of litter C, as calculated from Equation (1) in the main text. **(A)** Relations between e and the fraction of remaining C ($x = C/C_0$), colour coded according the value of the slope ε (as indicated in the legends on the right). **(B)** Relation between the time-derivative of the total litter C ($d(C/C_0)/dt$) and C/C_0, for different values of ε. **(C)** Temporal trajectories of C/C_0, for different values of ε. To solve Equation (1), a linear decomposition rate was assumed ($D = kC$), with first order kinetics constant set to $k = 1$ y^{-1} for illustration. Note that for $\varepsilon = 1$, the C-use efficiency at the beginning of decomposition is $e_0 = 1$, resulting in the unrealistic limiting case of no C loss.

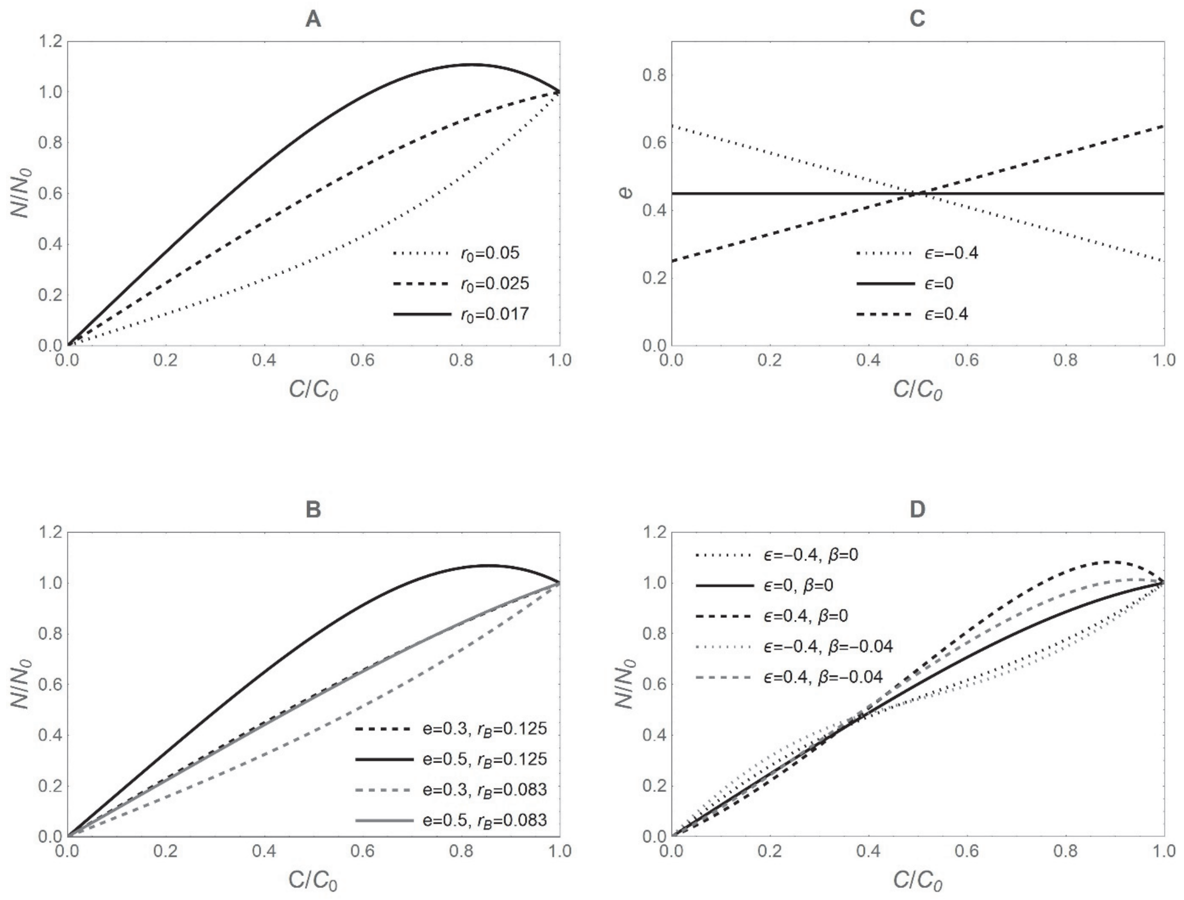

Figure S2. Effect of contrasting C-use efficiency parameterizations on modelled nitrogen release from decomposing litter, expressed by the fraction of remaining nitrogen ($y = N/N_0$) as a function of the fraction of remaining carbon ($x = C/C_0$) (equivalent to Figure 1 in the main text, except for α =2). Left panels show the predicted $y(x)$ for constant decomposer traits (model I) when varying: **(A)** litter N:C ratio, r_0, or **(B)** C-use efficiency (e) and decomposer biomass N:C ratio, r_B. **(C)** Assumed $e(x)$ relations (see Table 2 in the main text) and **(D)** corresponding $y(x)$ curves when r_B is either assumed time-invariant ($\beta = 0$; model II) or decreasing during decomposition ($\beta < 0$; model III). Unless otherwise specified in the legends of each panel, e =0.45, r_B =0.1 ($(C:N)_B$ =10), and r_0 =0.025 ($(C:N)_0$ =40); in all panels, α =2.

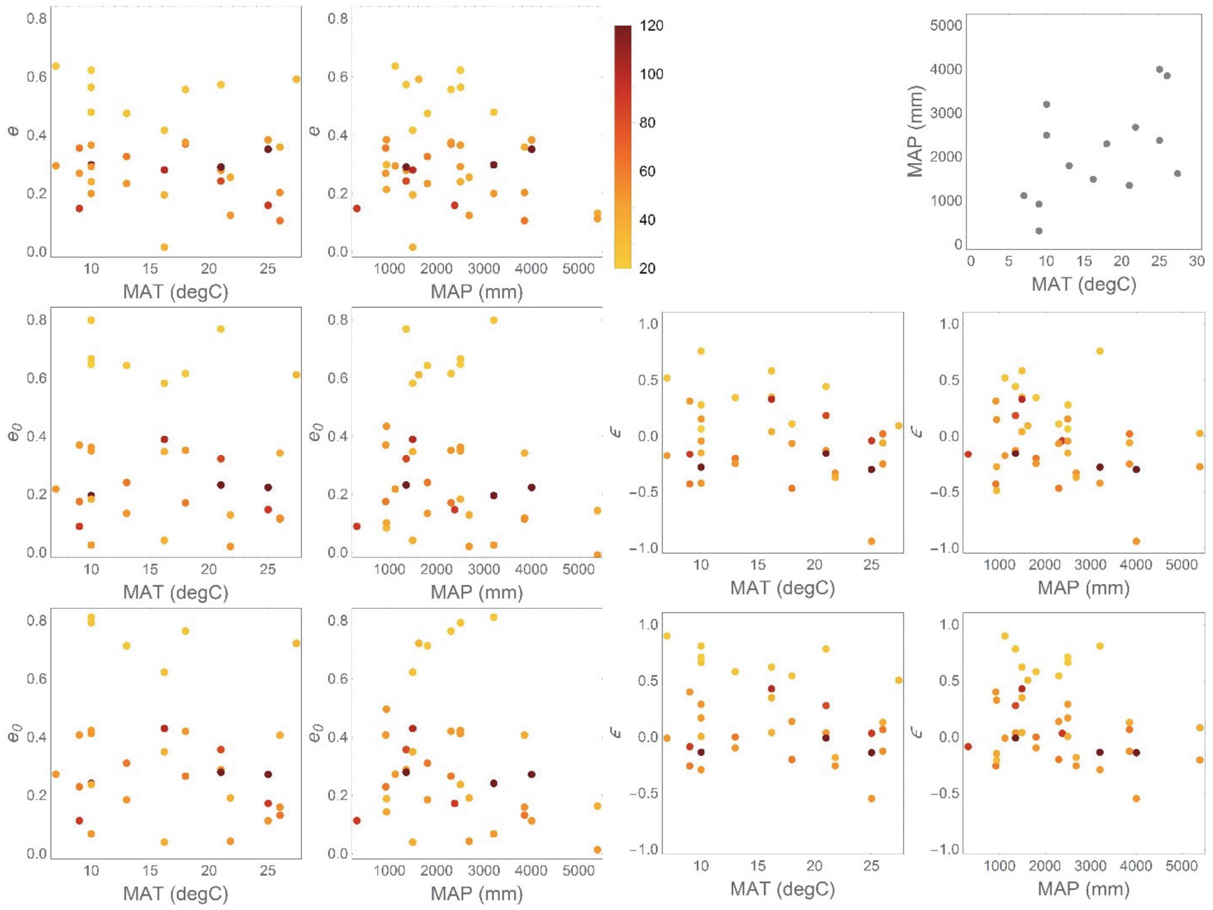

Figure S3. Dependence of the C-use efficiency parameters on mean annual temperature (MAT) and precipitation (MAP), for models I, II, and III (top, centre, and bottom rows, respectively; see details on the C-use efficiency models in Table 2 of the main text). Symbols are colour coded as a function of the litter initial C:N ratio, as indicated by the colour-bar. No regression lines between climatic conditions and C-use efficiency parameters are shown because none has slope significantly different from zero (at 95% confidence). The top right panel shows the combinations of MAT and MAP at the litter incubation sites (Table S1).

Reference list

Boberg, J.B., Finlay, R.D., Stenlid, J., Ekblad, A., Lindahl, B.D., 2014. Nitrogen and Carbon Reallocation in Fungal Mycelia during Decomposition of Boreal Forest Litter. PLOS One 9.

Chuyong, G.B., Newbery, D.M., Songwe, N.C., 2002. Litter breakdown and mineralization in a central African rain forest dominated by ectomycorrhizal trees. Biogeochemistry 61, 73-94.

Frankland, J.C., Lindley, D.K., Swift, M.J., 1978. Comparison of two methods for estimation of mycelial biomass in leaf litter. Soil Biology & Biochemistry 10, 323-333.

Harmon, M.E., 2005. LTER Intersite Fine Litter Decomposition Experiment (LIDET): Long-Term Ecological Research. Corvallis, OR: Forest Science Data Bank: TD023. http://www.fsl.orst.edu/lter/data/abstract.cfm?dbcode=TD023.

Hirobe, M., Sabang, J., Bhatta, B.K., Takeda, H., 2004. Leaf-litter decomposition of 15 tree species in a lowland tropical rain forest in Sarawak: dynamics of carbon, nutrients, and organic constituents. Journal of Forest Research 9, 347-354.

Isaac, S.R., Nair, M.A., 2005. Biodegradation of leaf litter in the warm humid tropics of Kerala, India. Soil Biology & Biochemistry 37, 1656-1664.

Kominkova, D., Kuehn, K.A., Busing, N., Steiner, D., Gessner, M.O., 2000. Microbial biomass, growth, and respiration associated with submerged litter of Phragmites australis decomposing in a littoral reed stand of a large lake. Aquatic Microbial Ecology 22, 271-282.

Lashermes, G., Gainvors-Claisse, A., Recous, S., Bertrand, I., 2016. Enzymatic Strategies and Carbon Use Efficiency of a Litter-Decomposing Fungus Grown on Maize Leaves, Stems, and Roots. Frontiers in Microbiology 7.

Osono, T., Takeda, H., 2004. Accumulation and release of nitrogen and phosphorus in relation to lignin decomposition in leaf litter of 14 tree species. Ecological Research 19, 593-602.

Osono, T., Takeda, H., 2005. Decomposition of organic chemical components in relation to nitrogen dynamics in leaf litter of 14 tree species in a cool temperate forest. Ecological Research 20, 41-49.

Tripathi, S.K., Singh, K.P., 1992. Nutrient Immobilization and Release Patterns During Plant Decomposition in a Dry Tropical Bamboo Savanna, India. Biology and Fertility of Soils 14, 191-199.

Tu, L.H., Hu, H.L., Chen, G., Peng, Y., Xiao, Y.L., Hu, T.X., Zhang, J., Li, X.W., Liu, L., Tang, Y., 2014. Nitrogen Addition Significantly Affects Forest Litter Decomposition under High Levels of Ambient Nitrogen Deposition. PLOS One 9.

Xu, X.N., Hirata, E.J., 2005. Decomposition patterns of leaf litter of seven common canopy species in a subtropical forest: N and P dynamics. Plant and Soil 273, 279-289.
